# A novel endoscopic approach for the treatment of hiatal hernia combined with refractory gastroesophageal reflux disease

**DOI:** 10.1055/a-2318-3222

**Published:** 2024-06-03

**Authors:** Yushang Yang, Xinyi Zhang, Kaihan Wu, Chencong Zhou, Xuan Huang

**Affiliations:** 1 74723 Department of Gastroenterology, The First Affiliated Hospital of Zhejiang Chinese Medical University, Hangzhou, China

Hiatal hernia (HH) is an important cause of refractory gastroesophageal reflux disease (GERD) 1 . Repair of hiatal hernias primarily relies on surgical intervention, with a lack of robust options for endoscopic treatments 2 . Here, we report a case in which a hiatal hernia combined with refractory GERD was successfully treated by gastric fundal and esophageal mucosal ligation combined with titanium clips (GEML-C).

A 69-year-old woman came to our hospital with a 20-year history of refractory GERD. Despite being on twice-daily proton pump inhibitor (PPI) therapy, she continued to experience symptoms, primarily acid reflux. The gastroscopic report suggested grade C erosive esophagitis and presence of a hiatal hernia ( Fig. 1 **a, b** ). Esophageal manometry confirmed a grade III hiatal hernia ( Fig. 2 ). The patient opted for GEML-C after the discussion of the options.

**Fig. 1:**
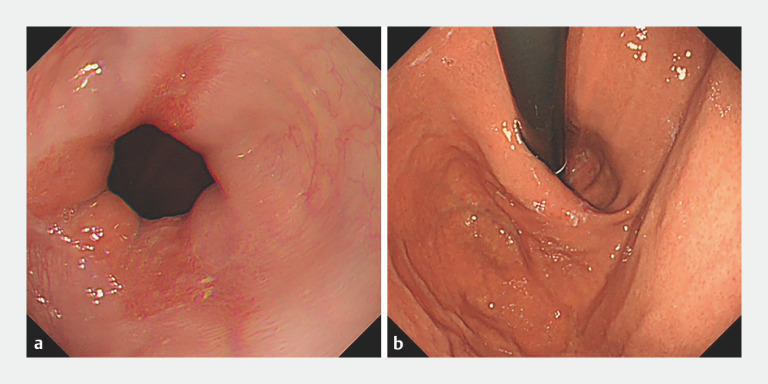
Endoscopic appearance before the procedure showing: **a** on forward-viewing gastroscopy, grade C erosive esophagitis at the esophagogastric junction; **b** with the gastroscope curved posteriorly to visualize the cardia and fundus of the stomach, a hernia sac of approximately 2.3 cm in length.

**Fig. 2:**
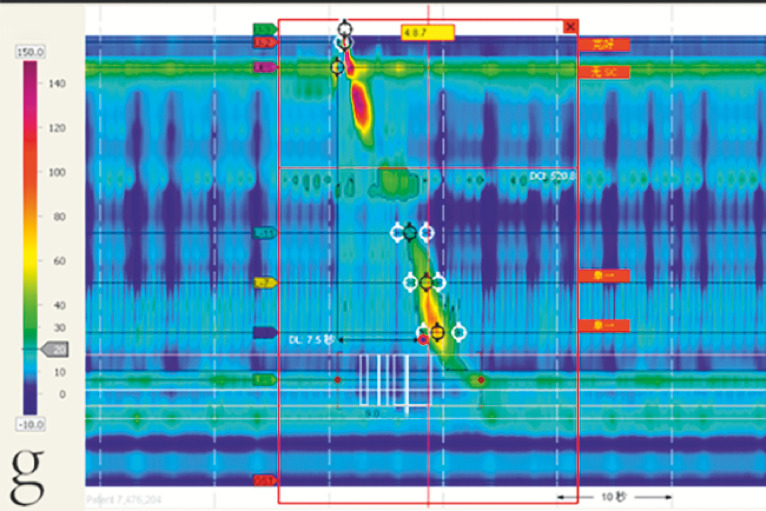
The patient’s preoperative esophageal manometry report.

A therapeutic endoscope was used throughout the whole process. With use of the inverted mirror condition, ligatures were placed with a multiring ligator (MBL-U-10; Cook Medical, USA) in a direction that was parallel to the angle of His. Six ligature rings were placed on the fundal side of the hernia sac ( Fig. 3 **a** ). Two ligation rings were placed in the lower esophagus on the sides of the greater and lesser curvatures, and two large titanium clips (ROCC-D-26-195; MT, China) were placed at the base of the ligations ( Fig. 3 **b** ). The lack of active resection in GEML-C is speculated to increase its safety profile, with a reduced risk of bleeding and perforation. In addition by ligating both the stomach and esophagus at the same time, it plays a better role in repairing the hernia sac and improving antireflux ( Video 1 ).

**Fig. 3:**
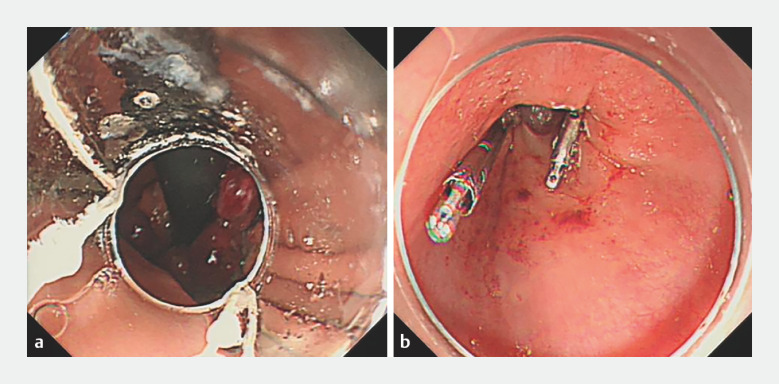
Endoscopic images of the gastric fundal and esophageal mucosal ligation combined with titanium clips (GEML-C) procedure showing: **a** six ligature rings placed on the fundal side of the hernia sac; **b** two ligature rings placed in the lower part of the esophagus on the sides of the greater and lesser curvature, with their bases clamped shut by placement of two large titanium clips.

A hiatal hernia combined with refractory GERD is successfully treated by gastric fundal and esophageal mucosal ligation combined with titanium clips (GEML-C), which includes the placement of six ligature rings on the fundal side of the hernia sac and two ligature rings in the lower part of the esophagus on the sides of the greater and lesser curvature, plus application of two large titanium clips at their bases.Video 1

The patient was discharged 1 day after the procedure. By 2 weeks later, she had reduced her dosage of PPI from twice daily to twice a week. After 3 months of follow-up, her clinical symptoms, gastroscopy, and esophageal manometry results all showed significant improvement ( Fig. 4 and Fig. 5 ; Table 1 ).

**Fig. 4:**
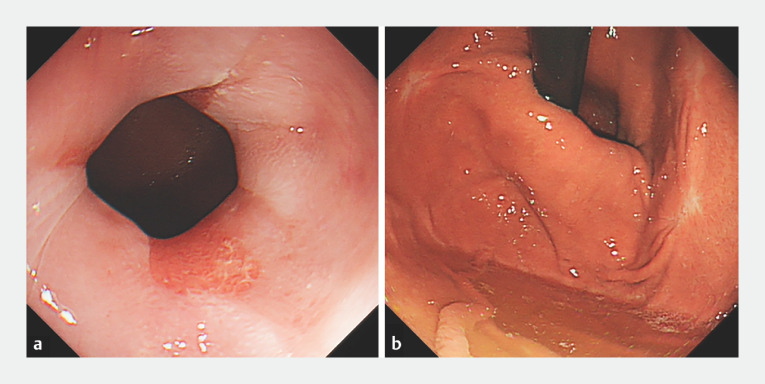
The endoscopic appearance 3 months after the procedure was completed showing: **a** grade B erosive esophagitis; **b** a smaller hernia sac with a length of about 1 cm.

**Fig. 5:**
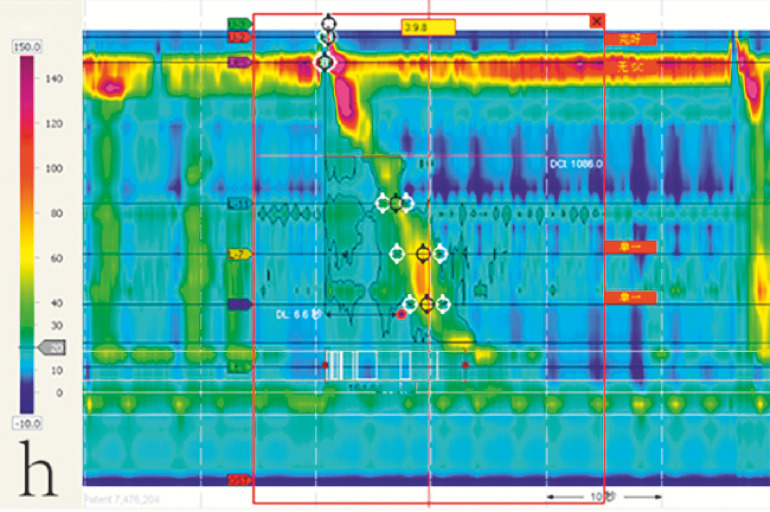
The patient’s postoperative esophageal manometry report.

**Table:** **Table 1** Pre- and post-procedural clinical data.

	Esophagitis grade (A–D)	Hiatal hernia size, cm	Hiatal hernia classification	Lower esophageal sphincter
Pre-procedure	C	2.3	Type III	Slack
Post-procedure	B	1.0	Type II	Not slack

This case suggests that this new type of minimally invasive endoscopic interventional therapy may be safer and faster for the treatment of hiatal hernia combined with refractory GERD.

Endoscopy_UCTN_Code_TTT_1AO_2AJ

## Notes

Endoscopy E-Videos https://eref.thieme.de/e-videos
*E-Videos* is an open access online section of the journal *Endoscopy* , reporting on interesting cases and new techniques in gastroenterological endoscopy. All papers include a high-quality video and are published with a Creative Commons CC-BY license. Endoscopy E-Videos qualify for HINARI discounts and waivers and eligibility is automatically checked during the submission process. We grant 100% waivers to articles whose corresponding authors are based in Group A countries and 50% waivers to those who are based in Group B countries as classified by Research4Life (see: https://www.research4life.org/access/eligibility/ ). This section has its own submission website at https://mc.manuscriptcentral.com/e-videos .
